# Supplementary material for: High-throughput image processing software for the study of nuclear architecture and gene expression
Source: Sci Rep. 2024 Aug 8;14:18426. doi: 10.1038/s41598-024-66600-1 (PMC11310328; doi:10.1038/s41598-024-66600-1)
Supplement: Supplementary file 1 — Supplementary Information. [file 41598_2024_66600_MOESM1_ESM.docx]

**Supplementary Note 1**

**Hardware Configuration**

For the computation and analysis of the datasets within this study, three high-performance computing systems were utilized. Two nodes were allocated from the NIH Biowulf HPC cluster, and one dedicated workstation was employed. The specifications for each system are detailed below.

*HPC Node 1:*

- GPUs: 4x NVIDIA Tesla V100 GPUs per node

- Scratch Space: 500 GB

- CPUs: 32 CPU cores per task, Intel(R) Xeon(R) CPU E5-2680 v4 @ 2.40GHz

- Memory: 120 GB of RAM per node

*HPC Node 2:*

- GPU: NVIDIA Tesla P100 (4x GPUs)

- Local Scratch Space: 400 GB

- CPU Cores: 32 per task, Intel(R) Xeon(R) CPU E5-2680 v4 @ 2.40GHz

- Memory: 120 GB

The nodes were requested using the Slurm Workload Manager with the `sinteractive` command, specifying the GPU (`--constraint=gpuv100`) and memory constraints, as well as the allocation of local scratch space and CPUs per task as shown.

*Local Workstation:*

The workstation used in this study was configured with the following hardware:

- Operating System: Ubuntu 18.04.6 LTS

- Memory: 251.6 GB

- Processor: AMD® Ryzen Threadripper 3970X 32-core processor x 64

- Graphics: Quadro RTX 8000/PCIe/SSE2

- OS Type: 64-bit

- Disk Space: 17.2 TB

**Table 1 Time required to load and visualize images with different number of channels**

| Visualization Time  (Seconds)  Hardware | One Channel | Two Channels | Three Channels | Four Channels |
| --- | --- | --- | --- | --- |
| HPC Node 1 | 0.32 | 0.59 | 0.86 | 1.16 |
| HPC Node 2 | 0.32 | 0.57 | 0.81 | 1.07 |
| Local Workstation | 0.14 | 0.25 | 0.35 | 0.48 |

Table 1 summarizes the processing times to load and visualize images from an example dataset with four channels using each one of the computational hardware mentioned above. Using the HiTIPS GUI we can select the number of channels we need to visualize at the same time. Each image is a 16-bit 2D array of size 1998×1998 pixels that takes up ~8MB space on the hard drive.

**Supplementary Note 2**

**Image Processing Algorithms**

## *Algorithm 1: CPU-Based Nuclei segmentation.*

1. The image $I$ is padded with a 5-pixel border of zeros.
2. The median filtering is applied to remove background noise while maintaining the nuclei edge:

$$I'(x)=median(I(N(x))$$

3. The image $I'$ is binarized by setting the threshold $T$ that is determined through Li's iterative method, which minimizes the cross-entropy of the two classes separated by the threshold:

$T=argmin\sum_{c_{1},c_{2}} p\left( c \right)\log\left( \mu_{c} \right)$.

1. The binary image $B(x)$ is filled with dilation and opening.
2. To connect the fragmented pieces of the nuclei, a Gaussian kernel $G$ is applied to the binary image $B(x)$ and then the intensities are scaled for thresholding:

$$B^{'}\left( x \right)=\left( G*B \right)\left( x \right)$$

3. Step 3 is repeated, and binary opening is applied to $B^{'}\left( x \right).$
4. The output image $D\left( x \right)$ is the Euclidean distance of each pixel to the nearest zero pixel in the binary image: $D\left( x \right)=\min_{y:B^{'}\left( y \right)=0} \left\| x-y \right\|$ and repeat step 3.
5. Connected components are labeled.
6. The center of mass `CM(i)` for each labeled region `i` is calculated as: $CM\left( i \right)=\frac{1}{N\left( i \right)}\sum_{x\in R\left( i \right)} x$
7. Create a new mask image $M\left( x \right)$ where $M\left( CM\left( i \right) \right)=1$ for each region $i$ and $M\left( x \right)=0$ otherwise.
8. The output image $W\left( x \right)$ is the watershed transform of the negative distance-transformed image -$D\left( x \right)$ using the marker image $M\left( x \right)$.
9. The boundary image $B\left( x \right)$ is defined where the labels in $W\left( x \right)$ change.
10. Resize the boundary image to the original size using interpolation and fill the holes in the boundary image to create the final mask.

*Algorithm 2: Intensity based timelapse nuclei alignment and RNA/gene assignment.*

Input**:** Time-lapse images with nuclei.

Output**:** Aligned nuclei and RNA/gene assignments.

Steps:

1. Initialization:

Set the nucleus major axis with the horizon at the initial time $t_{0}$:

$\alpha_{t_{0}}=0$

1. Setting default values for $\alpha\boldsymbol{'}$ and $\alpha\boldsymbol{"}$ :

- For each $t_{n} and n>0:$

$set {\alpha'}_{n}=0 and {\alpha"}_{n}=180$

1. Feature calculation:

Calculate the feature set $\mathcal{F}_{k} (k=1,\ldots, 8)$ for both ${\alpha'}_{n}=0 and {\alpha"}_{n}=180$:

1. Cosine Similarity Index
2. Mutual Information
3. Structural Similarity Index
4. Mean Square Error
5. Variation of Information
6. Adaptive Random Error
7. Peak Signal to Noise Ratio
8. Major axis decision:

If $\mathcal{N(F}_{k} \left( {\alpha^{'}}_{n}, \alpha_{n-1} \right)> \mathcal{F}_{k} \left( {\alpha^{”}}_{n}, \alpha_{n-1} \right)$ for more than 6 features

- Set $\alpha_{n}={\alpha^{'}}_{n}$. Otherwise:
- Set $\alpha_{n}={\alpha^{”}}_{n}$.

1. Fine-tuning of the major axis:

For *I* ranging from *1 to 5:*

- Set ${\alpha^{'}}_{n}=\alpha_{n}.$
- Set ${\alpha”"}_{n}=\alpha_{n}\pm i$.

If $\mathcal{N(F}_{k} \left( {\alpha^{'}}_{n}, \alpha_{n-1} \right)> \mathcal{F}_{k} \left( {\alpha^{”}}_{n}, \alpha_{n-1} \right))$ for more than 6 features, then

- Set $\alpha_{n}={\alpha^{'}}_{n}$.
- Increment $n$ by 1 and exit the loop. Otherwise:
- Set $\alpha_{n}=”_{n}$.

1. Mapping and tracking:

- Map spot coordinates to nuclei patch.
- Track the spots using Algorithms 5 and 6

*Algorithm 3: Phase-transform timelapse nuclei alignment and RNA/Gene assignment*

Inputs:

- Reference image $R$
- Image to be registered $I$
- Initial rotation angle $\alpha_{t_{1}}$
- Rescale factor $F$
- Median filter size ${Disk}_{rad}$
- Nuclei length $L$

Outputs:

- Final rotation angle $\alpha_{final}$
- Aligned image $I_{aligned}$

Steps:

1. Initialization:

If no $R$ exists, set $R=I$ and return; set $\alpha_{t_{1}}=15$

1. Pre-processing:

- For both $R$ and $I$:

$$R_{rescale}=Rescale(MedianFilter\left( R,{Disk}_{rad} \right))$$

$$I_{rescale}=Rescale(MedianFilter\left( I,{Disk}_{rad} \right))$$

1. Polar transformation:

$$radius=L\times F$$

$$R_{polar}=WarpPolar(R_{rescale}, radius)$$

$$I_{rot}=Rotate\left( I_{rescale},\alpha_{t_{1}} \right)$$

$$I_{polar}=WarpPolar(I_{rot}, radius)$$

2. Cross-correlation in polar Fourier domain:

$$\left( \Delta x,\Delta y \right), error, phasediff=PhaseCrossCorrelation(R_{polar}, I_{polar})$$

$$\alpha_{\mathrm{final}}=-\Delta y$$

3. Rotation Correlation:

$$\alpha_{corrected}=\alpha_{final}-\alpha_{t_{1}}$$

$$I_{aligned}=Rotate\left( I,\alpha_{corrected} \right)$$

4. RNA/Gene assignment:

$$Spot Coordinates=MapToNucleiPatch(I_{aligned})$$

$$Track spots using Algorithms 5 and 6$$

5. Return:

$$\mathrm{Return}\alpha_{\mathrm{final}}, I_{\mathrm{aligned}}$$

## *Algorithm 4: Hierarchical clustering and outlier removal algorithm.*

## Inputs:

- A set of $n$ data points $P=\{p_{1}, p_{2}, \ldots, p_{n}\}$
- Outlier threshold $T_{out}$
- Maximum distance $D_{max}$

Steps:

1. Compute the pairwise Euclidean distance matrix $D =\left[ d_{ij} \right]$ , where $d_{ij}= \sqrt{\sum_{i,j\in\{1,2,\ldots,n\}} {(p_{i}-p_{j})}^{2}}$
2. Perform single-linkage hierarchical clustering on the distance matrix to generate an initial set of labels $L$.
3. Identify the unique labels $U=\{u_{1}, u_{2}, \ldots, u_{m}\}$ and calculate their centroids $C=\{c_{1}, c_{2}, \ldots, c_{m}\}$, where $c_{k}=\frac{1}{\left| \{i:l_{i}=u_{k}, i\in\left\{ 1,2,\ldots,n \right\}\} \right|}\sum_{l_{i}=u_{k}}^{n} p_{i}$
4. Calculate the standard deviation $S=\{s_{1}, s_{2}, \ldots, s_{m}\}$ of distances from the centroid for each cluster $s_{k}=\sqrt{\frac{1}{\left| \{i:l_{i}=u_{k}, i\in\left\{ 1,2,\ldots,n \right\}\} \right|}\sum_{l_{i}=u_{k}}^{n} {(d_{ik}-\bar{d_{k}})}^{2}}$
5. Initialize an array $outlier\_index$ of zeros with size $n$.
6. For $k=1, \ldots, m$:
   1. Compute the Euclidean distances $d_{ik}$ of all points $p_{i}$ in the cluster to the centroid $c_{k}$.
   2. Identify outliers $O_{k}$ as those points where $d_{ik}> T_{out}*D_{max}$.
   3. Update the corresponding indices in $outlier\_index$ for the outliers: $outlier\_index[i]=1; if p_{i}\in O_{k}$.
7. Assign a label indicating outliers (usually 0) to points identified as outliers in the initial set of clusters $l_{i}=0, if outlier\_index[i]=1$.
8. Apply the "Merge Small Clusters Algorithm" to merge small clusters and outliers.
9. Return the final set of cluster labels $L$.

*Algorithm 5: Merge small clusters algorithm.*

Inputs:

- A set of $n$ data points $P=\{p_{1}, p_{2}, \ldots, p_{n}\}$
- Corresponding labels $L=\{l_{1}, l_{2}, \ldots, l_{n}\}$
- A maximum distance $D_{max}$
- Minimum size threshold $T$

Steps:

1. Calculate the size of each cluster $s_{i}$ for $i=1, 2, ...,c$,$s_{i}=\sum_{j=1}^{n} \delta_{l_{j,i}}$ ($\delta is Kronecker delta function, which is equal to 1 if l_{j}=i and 0 otherwise)$
2. Define $large\_clusters=\{i : s_{i}\geq T\}$ and $samll\_clusters=\{i : s_{i}<T\}$.
3. Identify the data points $p_{large}$ belonging to $large\_clusters$.
4. For each $i$ in $samll\_clusters$, find the indices of points $I_{i}$ in $P$ belonging to that cluster $I_{i}=\left\{ j \right| I_{j}=i, for j=1, 2,\ldots, n\}$.
5. For each point $p_{j}$ in $I_{i}$ for $i$ in $samll\_clusters$:
   1. Compute the Euclidean distances $d_{kj}$ to all points in $p_{large}$, where $d_{kj}= \sqrt{\sum_{k\in p_{large}} {(p_{j}-p_{k})}^{2}}$
   2. Find $m_{k}=argmin(d_{kj})$, the point in $p_{large}$ with the smallest distance to $p_{j}$.
   3. If $d_{mj}\leq D_{max}$, then assign $l_{j}=l_{m}$, where $l_{m}$ is the label of $m_{k}$.
   4. Otherwise, assign $l_{j}=0$.
6. Return the updated labels $L$.

## *Algorithm 6: Local background estimation algorithm.*

Input:

- An image represented by a 2-dimensional matrix $I\in\mathcal{R}^{m*n}$ , where $m$ and $n$ are the image's height and width, respectively.

Steps:

1. Initialize two empty arrays $x_{border}\in\mathcal{R}^{2m}$ and $y_{border}\in\mathcal{R}^{2n}$ to store the border pixel intensities.
2. Fill the first half of $x_{border}$ with the first column of $I$, and the second half with the last column of $I$. Similarly, fill the first half of $y_{border}$ with the first row of $I$, and the second half with the last row of $I$.
3. Create two arrays $x\in\mathcal{R}^{2m}$ and $y\in\mathcal{R}^{2n}$, each containing indices ranging from $0 to m-1$ and $0 to n-1$ respectively, repeated twice.
4. Compute the least squares fit parameters for both axes following Bevington's method:
   - Compute $\Delta x=2m(\sum x^{2}-{(\sum x)}^{2})$ and $\Delta y=2m(\sum y^{2}-{(\sum y)}^{2})$ .
   - Compute the fit parameters $a=\frac{(\sum x^{2}\sum x_{border}-\sum x\sum x_{border})}{\Delta x}$ and $b=\frac{(2m\sum x*x_{border}-\sum x\sum x_{border})}{\Delta x}$ for the x-axis.
   - Similarly, compute the fit parameters $c=\frac{(\sum y^{2}\sum y_{border}-\sum y\sum y_{border})}{\Delta y}$ and $d=\frac{(2m\sum y*y_{border}-\sum y\sum y_{border})}{\Delta y}$ for the y-axis.
5. Compute the plane's offset: $offset=\frac{\left( a-\frac{d\left( n-1 \right)}{2}+c-\frac{b\left( m-1 \right)}{2} \right)}{2}$
6. Generate a plane$P\in\mathcal{R}^{m*n}$ using the offset and slope parameters computed. For every pixel $(i,j)$ in $P$, set $P\left( i,j \right)=offset+b*i+d*j$ .
7. Return $P$ as the local background of $I$.

Note: This method uses the least squares method to fit the border pixel intensities to a plane. The offset of the plane is averaged over each edge in x, and each edge in y. The actual offset is corrected for the tilt of the plane. Then, the two offsets are averaged together to give a single offset.

## *Algorithm 7: Gaussian mask fitting algorithm.*

Input:

- An image is represented by a 2-dimensional matrix $I\in\mathcal{R}^{m*n}$ where $m$ and $n$ are the image's height and width, respectively.
- An optional point ${xy}_{input}=[x_{input}, y_{input}]$ to initialize the centroid.
- A Boolean variable $fit$ to decide if the fitting process should be applied.

Steps:

1. Initialize $x_{0}$ and $y_{0}$ as $0$. $0$ to represent the initial centroid's coordinates. Initialize the Gaussian mask $G\in\mathcal{R}^{m*n}$ as a zero matrix.
2. Compute a scalar factor $F=\frac{1}{\sigma\sqrt{2}}$ .This is a constant specific to the Gaussian Mask (GMASK) fitting process, where $\sigma$ is the width of the Gaussian PSF of the imaging system.
3. Compute the local background $B$ of the image $I$ and subtract it from $I$ to get $I_{no\_bg}$. Any negative values in $I_{no\_bg}$ are set to $0$.
4. Set the border of $I_{no\_bg}$ to $0$. This is a boundary condition applied to ensure the mask doesn't bleed over the edges of the image.
5. If $fit$ is $False$, assign $x_{0}$ and $y_{0}$ as $x_{input}$ and $y_{input}$ respectively. Compute the Gaussian mask $G$ using the error function $erf$ as follows:
   - For each pixel $(x, y)$ in $G$, compute four quantities: $a=F*(y-0.5-y_{0})$, $b=F*(y+0.5-y_{0})$, $c=F*(x-0.5-x_{0})$, and $d=F*(x+0.5-x_{0})$.
   - Set $G\left( x, y \right)=0.25\left( \mathrm{erf} \left( a \right)-\mathrm{erf} \left( b \right) \right)*\left( \mathrm{erf} \left( c \right)-\mathrm{erf} \left( d \right) \right)$ .
6. If $fit$ is $True$, apply the iterative centroid calculation with the Gaussian mask. Until the difference between the old and new centroids is less than a small threshold $h={10}^{-3}$, or after a maximum of 300 iterations:
   - Update the centroid $x_{0}$ and $y_{0}$ by adding half of ${diff}_{x}$ and ${diff}_{y}$ respectively.
   - Compute the Gaussian mask $G$ as described in step 5.
   - Calculate the sums of pixel values in $I_{no\_bg}$ multiplied by $G$, and calculate the sums of pixel coordinates multiplied by $I_{no\_bg}$ and $G$. Let these be $sum\_$, ${trial}_{x_{0}}$, and ${trial}_{y_{0}}$.
   - Update ${diff}_{x}$ and ${diff}_{y}$ as (${trial}_{x_{0}}$ / $sum\_$ - $x_{0}$) and (${trial}_{y_{0}}$ / $sum\_$ - $y_{0}$).
7. Return $results$ which contains the final centroid coordinates and the estimated photon number.

**Supplementary Note 3**

|  | Visualization | Nuclei Segmentation | Spot detection | Nuclei Tracking | Nuclei Registration | Spots Tracking |
| --- | --- | --- | --- | --- | --- | --- |
| HiTIPS | Yes | Yes | Yes | Yes | Yes | Yes |
| CellProfiler | Yes | Yes | Yes | Yes | No | No |
| ImageJ | Yes | Yes | Yes | Yes | No | No |
| FISH-Quant V2 | Yes | Yes | Yes | No | No | No |
| Tonga | Yes | Yes | No | No | No | No |
| Napari | Yes | No | No | No | No | No |
| MoBie | Yes | No | No | No | No | No |

**Supplementary Note 4**

**HiTIPS Analysis Pipeline Architecture**

The software architecture of HiTIPS is organized in Python classes that, on a per FOV basis, run image processing functions, save intermediate results as serialized Python objects on disk, and maintain state information on the progress of the image analysis pipeline for their specific part of the analysis. In particular, the core of a HiTIPS pipeline is the BatchAnalysis class, which calls specific image processing methods from each image analysis class (e.g. Nucleus Segmentation class) based on the analysis parameters in the proper order, checks on the progress of the image processing step, and saves the state of the analysis workflow (Analysis pipeline progress, location of the intermediate results on disk, etc.), and reads and aggregates the intermediate results of the analysis pipeline when all the FOVs have been analyzed.

The BatchAnalysis class starts the analysis pipeline by initializing the required environment and configurations, includes defining file read and write paths on the file system, reading image acquisition metadata (Well, Field, Time point, z-plane, channel indexes), preparing intermediate data structures, and, most importantly, reading and saving the order of the different image analysis tasks in the pipeline as defined by user input parameter configuration. Once the initial setup is complete, the BatchAnalysis class proceeds to the actual image analysis pipeline, whose first step involves the sorting of individual images in processing batches on a per FOV basis based on the acquisition metadata. At this point, the BatchAnalysis class launches separate parallel processing threads (Workers), each one tasked to analyze a batch of images belonging to a single FOV. The number of processes to be run in parallel at any given time is a user-defined parameter that depends on the number of available CPUs on the computer used to run the HiTIPS analysis.

Each worker process begins the analysis by calling specific analysis methods from the appropriate image analysis class (e.g. Nucleus Segmentation, Spot Detection, etc). At the end of the method call, intermediate results are saved to disk, intermediate image masks are saved to disk (Only if the user selects this option), and the BatchAnalysis class is updated to reflect the completion of the analysis method for the particular process, and to save the identity and location of the intermediate results. Once this update is completed, and based on the input parameters that specify the order of the different image analysis classes in the pipeline, the BatchAnalysis class calls the next set of specific analysis methods from the next image analysis class. For each process, this sequence of events is repeated until all the image analysis steps in the pipeline have been completed. At this point, the BatchAnalysis class can initiate another process to analyze another remaining batch of images from another FOV. Finally, when all the FOVs in the dataset have been analyzed, the BatchAnalysis class reads all the serialized intermediate results from disk, aggregates the results on a per well basis, and saves both the per object and the per well results as csv files.
